# Knowledge and Attitudes Regarding Non-Invasive Prenatal Testing (NIPT) and Preferences for Risk Information among High School Students in Sweden

**DOI:** 10.1007/s10897-016-9997-y

**Published:** 2016-08-02

**Authors:** Susanne Georgsson, Ellika Sahlin, Moa Iwarsson, Magnus Nordenskjöld, Peter Gustavsson, Erik Iwarsson

**Affiliations:** 1 Sophiahemmet University, Box 5605, SE-114 86 Stockholm, Sweden; 2 Department of Clinical Science, Intervention and Technology, Karolinska Institutet, Stockholm, SE-171 77 Sweden; 3 Department of Molecular Medicine and Surgery, Karolinska Institutet, Karolinska University Hospital, Solna, CMM (L8:02), SE-171 76 Stockholm, Sweden

**Keywords:** Prenatal testing, NIPT, Attitudes, Preferences, Risk information

## Abstract

Non-invasive prenatal testing (NIPT) was recently introduced for prenatal testing of genetic disorders. Cell-free fetal DNA is present in maternal blood during pregnancy and enables detection of fetal chromosome aberrations in a maternal blood sample. The public perspective to this new, simple method has not been illuminated. The views of young people (i.e. future parents) are important to develop suitable counseling strategies regarding prenatal testing. The aim was to explore Swedish high school students’ attitudes, knowledge and preferences regarding NIPT. A questionnaire was completed by 305 students recruited from one high school in Stockholm, November and December 2014. Most students (80 %) considered prenatal testing as good. The majority (65 %) was positive or very positive towards NIPT and 62 % stated that they potentially would like to undergo the test if they or their partner was pregnant. The vast majority (94 %) requested further information about NIPT. Most students (61 %) preferred verbal information, whereas 20 % preferred information via the Internet. The majority of the high school students was positive towards prenatal testing and most was positive towards NIPT. Further, information was requested by the vast majority before making a decision about NIPT. Most of the students preferred verbal information and to a lesser extent information via the Internet. The attitudes, knowledge and preferences for risk information concerning NIPT in young adults are important, in order to increase knowledge on how to educate and inform future parents.

## Introduction

Prenatal testing is an expansive field, and methods have improved dramatically in the past few years. The most recently implemented method is non-invasive prenatal testing (NIPT) for analysis of fetal aneuploidy. In a blood sample from the pregnant woman, circulating fetal cell free DNA (cfDNA) is present and can be used for analysis (Lo et al. 1997). The weighted pooled detection rates for trisomy 21, 18 and 13 using NIPT are 99.2 %, 96.3 %, and 91.0 %, respectively, with false-positive rates of 0.09 % for trisomy 21 and 0.13 % for trisomy 18 and 13 (review in Gil et al. 2015). The use of NIPT substantially reduces the need for invasive procedures (chorionic villus biopsy, amniocentesis) which are associated with an increased risk of miscarriage of around 0.1–0.5 % (Akolekar et al. 2015 Simpson 2012). NIPT is an easy, safe and early method (de Jong et al. 2010), and women have a strong preference for tests without a risk of miscarriage (Hill et al. 2012). Still, there is a question about how to best implement it in clinical practice (Deans and Newson 2012).

Previous studies have shown that public attitudes towards NIPT in the UK are positive (Kelly and Fairmond 2012) but the rapid development of methods for prenatal testing raises questions about the expectant parents’ ability to make informed choices (Björklund et al. 2012). When introducing this new prenatal screening method, possible risks have been discussed, such as that the simplicity of the test may be a threat to informed choice (de Jong et al. 2010,Vanstone et al. 2014). Other supposed dilemmas are due to the possibility that the test can be performed in early pregnancy: there might be limited time for decision-making about whether or not to undergo the test, the pregnant woman may accept the offer without reflecting, and the new technique may allow prenatal testing for a broader range of abnormalities than is possible in current procedures for the detection of prenatal aneuploidy, such as the first trimester combined test (FCT) (de Jong et al. 2010). There may be a risk for sex-selection and expanded chromosome analysis (Hill et al. 2012; Vanstone et al. 2014) as well as the risk that both testing and termination of fetuses with chromosome aberrations become”normalized” (de Jong et al. 2010; Farrimond and Kelly 2011). Extended counseling compared to current practices is necessary to facilitate decision-making (Vanstone et al. 2014).

Many aspects have to be covered in the information provided about prenatal testing (SOSFS 2012) and information about risks is complex (Austin 2010). The perception of risk varies, depending on background, socio-economic status, previous experiences, knowledge, worry and gender (Slovic et al. 2004), as well as the perception of context and severity of the potential outcome (Austin 2010). There are three main formats used to communicate risks: verbally, numerically or graphically. However, there is lack of knowledge about which format is the best. People often have a binary perception of risk; either the event is going to happen or not, and this way of considering risk is even more common when the outcome involves emotions (Timmermans 2005). It is difficult for pregnant women to understand and to interpret the number 1/X (e.g., a risk of 1/345), which often is given in the context of prenatal testing (Pighin et al. 2011). There is a need for the development of tools for risk communication during pregnancy (Keller and Siegrist 2009).

Informing patients about prenatal testing in general and risk information in particular, is challenging for the health professionals in antenatal care. In Sweden, parents-to-be are counseled by midwives except for more complex cases when further information is required, whereupon the couple will see a doctor. Little is known about the attitudes, levels of knowledge, preferences regarding NIPT and preferences for information among pregnant women, as well as young people and society in general. The new technology has been subject to debate in the research community but the public perspective has not been extensively illuminated. It is important to be aware of the views of young people (i.e., parents-to-be) in order to develop suitable strategies for future counseling regarding prenatal testing.

The aim of this study was to explore Swedish high school students’ attitudes, knowledge and preferences regarding Non-Invasive Prenatal Testing.

## Methods

### Participants

The study group consisted of 305 students, between 16 to 18 years of age, recruited from one high school in Stockholm. The students were recruited from different programs, but most attended either a natural science or a social science program. Data were collected by means of a two page questionnaire. The students were recruited from all three different grades in high school. For practical reasons, as well as for protecting confidentiality it was neither possible to record the non-participation rate nor the reasons for declining participation. The period for data collection was between 20 November and 10 December 2014 (i.e. before NIPT was available in Swedish healthcare).

### Instrumentation and Procedures

The 14-item questionnaire was developed by the research group. The questionnaire included demographic items about age and gender, and four items used in previous studies (van den Berg et al. 2005), that asked about attitudes towards having a prenatal test (answered on a five-points rating scale). Most of the questions were of Likert-type, with four- five- or six-point rating scales. Some of the questions had forced-choice answers and some stated alternatives to obtain a broad picture of the students’ attitudes, knowledge and presumptive decision-making. The questionnaire took approximately five minutes to complete. In order to collect the greatest possible range of answers, it was a prerequisite that the questionnaire was easy and quick to complete. The questionnaires were distributed by one of the co-authors (MI). Verbal information about the study, but not about the testing methods including NIPT, nor about chromosomal aberrations, was given to all participants. The students completed the questionnaires in the classroom and returned them immediately.

### Data Analysis

Statistical analyses were performed using the Statistical Package of Social Science (SPSS) version 23.0 for Windows (PSS Inc., Chicago, IL, USA). To calculate proportions of answers to questionnaire items, descriptive statistics (frequencies) were used. To calculate differences between genders chi-square tests were used, and the significance level was set to *p* < 0.05.

### Ethical Considerations

Participation was voluntary, and the students had the option to withdraw from the study by not handing in the questionnaire. The Swedish legislation on ethical considerations in research (18 §) states that a child aged fifteen, but not eighteen, who understands the aim of the study is able to give informed consent to participate (Law on ethical review of research involving humans 2003). The questionnaire was filled in anonymously. The Regional Ethical Review Board in Stockholm approved the study (DNR 2014/1817–31/4).

## Results

### Sample Demographics

There were 143 (46.9 %) students from the first grade, 55 (18.2 %) from the second, and 107 (35.1 %) students from the third grade. The sample’s mean age was 17 years. Of the 305 participants, 187 (62.5 %) were women, 107 (35.8 %) were men, and five students (1.7 %) stated their gender as “Other.”

### Attitudes towards Prenatal Testing

The students rated their attitudes towards prenatal testing on a five- point scale with the following attitudes stated as anchors: Good (1) – Bad (5); Frightening (1) – Not frightening (5); Not calming (1) – Calming (5); and Not self-evident (1) – Self-evident (5). Their responses were presented as three values (1–2), (3) and (4–5) where 3, was considered as a neutral attitude. (Table 1). Most of the students (80 %) perceived prenatal testing as good (i.e. answered 1–2 on the scale). More than half (55 %) perceived prenatal testing as “Not frightening,” and 49 % answered that the testing would be calming. Further, 41 % responded with ratings indicating it is self-evident that prenatal testing detects fetal abnormalities (Table 1). Regarding the question; “If you or your partner were pregnant and NIPT was available, would you undergo NIPT?” Sixty-two percent of the students reported they would potentially undergo NIPT (Score 4–5, where a 5 means “Yes, for sure”) whereas 10 % stated that they would not undergo such a test (Score 1–2, where a 1 means “No, absolutely not”).

**Table 1:** Students’ attitudes toward prenatal testing in general (*N* = 305)

	Score 1–2	Score 3	Score 4–5	
	*n* (%)	*n* (%)	*n* (%)	
Good	242 (80)	53 (18)	8 (3)	Bad
Frightening	69 (23)	65 (22)	164 (55)	Not frightening
Not calming	40 (14)	112 (38)	144 (49)	Calming
Not self-evident	73 (25)	100 (34)	121 (41)	Self-evident

*n*’s very slightly as not all students answered every item

The students stated their attitude towards different prenatal testing methods, on a scale ranging from 1 “Very negative” to 5 “Very positive.” If the testing method was unknown to the student, it was possible to indicate that as well. Their responses were presented as three values (1–2 “Very negative” or “Negative”), (3 “Neither”), and (4–5 “Positive” or “Very Positive”). For 30 %, FCT was unknown, and for 29 %, invasive tests were unknown. However, very few did not know about ultrasound examination (5 %). Almost one fifth, 17 %, did not know about NIPT. Attitudes towards ultrasound examination were rated as positive or very positive by 90 % of the students. More than two thirds, (65 %) were positive or very positive towards NIPT (Table 2). Of those 65 % of students (i.e., those who answered 4–5 on the scale), 79 % stated that they would be willing to pay for NIPT for themselves if the cost was not covered by the national health system. The majority (58 %) was willing to pay between 50 and 200 Euro, 15 % were willing to pay 500 Euro, and 2 % were willing to pay as much as 5000 Euro.

**Table 2:** Students’ attitudes toward different methods (*N* = 305)

	Do not know	Very negative - Negative	Neither negative nor positive	Positive – Very positive
		1–2	3	4–5
	*n* (%)	*n* (%)	*n* (%)	*n* (%)
CUB	91 (30)	1 (0.3)	28 (9)	180 (60)
Invasive test	86 (29)	10 (3)	61 (20)	143 (48)
Ultrasound	14 (5)	-	17 (6)	268 (90)
NIPT	51 (17)	9 (3)	44 (15)	191 (65)

n’s vary slightly as all students answered every item

### Attitudes towards Chromosome Aberrations

Sixty-three percent of the students reported that it would be of great significance to have a baby with Down syndrome, whereas 4 % stated that it would not be of great significance. The most frequent answer was 4 (selected by 41 %) on the scale where 5 was the highest – of very great significance. The highest score (5), was endorsed by 22 % of the students.

### Risk Perception

**Fig. 1:**
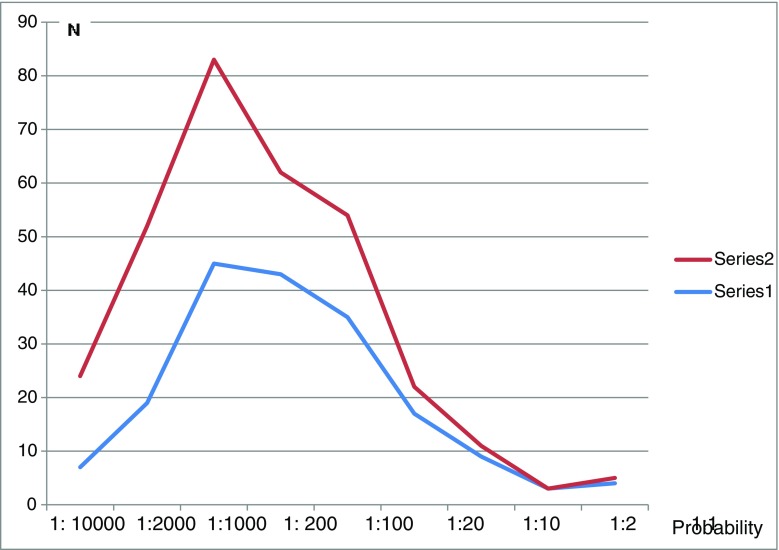
Perception of high probability to have a baby with a chromosomal aberration (red *n* = 182) and perception of own risk of having a baby with a chromosomal aberration (blue *n* = 134)

Students’ perceptions of the probability of having a baby with a chromosome aberration were assessed on a scale from 1:10,000 to 1:1. Some students (12 %) endorsed a value outside the given values and their responses were therefore excluded from this analysis. The sample also estimated their own probability of having a baby with a chromosome aberration on the same scale. The students estimated that the population in general had a higher probability to have a baby with chromosomal aberration compared to their own probability. The results are presented in Fig. 1.

### Knowledge about NIPT

Most of the students (62 %) did not know about NIPT before they completed the questionnaire. If NIPT was available, 62 % reportedly would want to undergo the test if they or their partner were pregnant (i.e., stated 4–5 on a scale ranked from 1 “No, absolutely not” to 5 “Yes, I am totally sure”). The most frequent answer on the scale was 4 (34 %). Ten percent endorsed 1–2 on the scale, indicating they would not want to undergo the test.

### Information about NIPT

The vast majority of the sample (94 %) reported they would need further information before making a decision about NIPT. Regarding how they would prefer to receive the information, the students were able to select more than one option. Sixty-one percent indicated verbal information from the midwife in antenatal care, whereas a separate visit to a midwife or doctor was endorsed by 58 %. Written information was preferred by 49 %. Twenty percent endorsed a preference for information via the Internet. A few students (3 %) selected “Other,” suggesting that this kind of information should be available in different ways. Six students provide a written comment; four of them wanted information from several sources, one student wrote that it does not matter, and another that information about the reactions of their partner would be crucial.

### Preferred Information from NIPT

More than half of the students reported they did not want to know the sex of the baby (54 %). However, the majority (77 %) wanted to know if the baby had Down syndrome, and 87 % if the baby had a more severe chromosome aberration. Two hundred students (66 %) indicated that they wanted to know if the baby had any chromosome aberration.

Table 3 contains a comparison of information preferences for those students who stated they would undergo NIPT (*n* = 182) and those who would not undergo NIPT (*n* = 26). A significantly smaller percentage of student in the “No NIPT” group preferred to know information about whether the fetus had Down syndrome, X^2^ (1) = 94.7, *p* < 0.00 l; if the fetus had a more severe chromosome abnormality, X^2^ (1) = 48.5, *p* < 0.00 l; or information about all detectable chromosome abnormalities, X^2^ (1) = 48.5, *p* < 0.00 l.

**Table 3:** Preferred information of students undergoing NIPT versus those not undergoing NIPT

Preferred information if undergoing NIPT	Students who would potentially undergo NIPT *(n* = 182) *n* (%)	Students who would not undergo NIPT (*n* = 26) *n* (%)	*p*-value
Fetal gender	69 (38)	9 (35)	0.608
If the fetus has Down syndrome	168 (92)	9 (35)	<0.001
If the fetus has a more severe chromosomal abnormality	174 (96)	19 (73)	<0.001
All detectable chromosomal abnormalities	154 (85)	6 (23)	<0.001

### The Decision about Prenatal Testing

Table 4 contains the answers to two questions regarding the important influences on students’ decisions to undergo prenatal testing and who would influence their decision. The most prevalent influence, stated by 89 %, was worry about the health of the baby, followed by a wish to know as much as possible, which was stated by 39 %. Approximately one fourth (23 %) wanted to undergo prenatal testing due to personal experiences of someone with a chromosome aberration or a severe disease. In addition to the given alternatives, the student had the possibility to fill in “Other” with a comment, which was provided by 34 students. Other influences mentioned frequently were the possibility to prepare for something being wrong with the baby and to increase the possibility to take care of the baby in the best way. Regarding *who* would influence the decision, the most frequent answer was the partner (83 %). Almost half (44 %), of the students stated that family and friends would influence the decision. About one fourth (26 %) stated that no one except themselves would influence the decision. Nine students filled in “Other” and of those, half stated that the decision would be taken by themselves.

**Table 4:** Important influences on students‘ decision about prenatal testing

What influences the decision about prenatal examinations?	
*n* = 302	***n (%)***
Worry about the health of the baby	268 (89)
I want to know as much as possible	117 (39)
No reasons to refuse	81 (27)
Personal experience of chromosomal aberration or other severe disease among relative	69 (23)
The values of the society	44 (15)
Expectations from others	41 (14)
Everyone else does	14 (5)
Important to know the gender of the baby	4 (1)
Who influences the decision about prenatal examinations?	
*n* = 302	
My partner	250 (83)
The doctor in antenatal care	168 (56)
Family and friends	132 (44)
The midwife in antenatal care	129 (43)
No one, I make the decision myself	80 (26)

*More than one response was possible for each of these questions

### Gender Differences

For two of the questions there were significant differences in response frequencies between women (*n* = 187) and men (*n* = 107). First, a greater percentage of the women (94 %) reported a positive or very positive towards ultrasound examination, compared to the men (83 %), X^2^ 16.6 , *p* = 0.001. Second, there was a significant gender difference concerning who influences the decision of whether or not to undergo prenatal testing; 32 % of the women reported they would make the decision themselves, compared to 19 % of the men, X^2^ 6.2, *p* = 0.01.

## Discussion

In the present study, high school students were studied in order to form a picture of young peoples’ attitudes, knowledge and preferences before they have reached childbearing age, without experience of being pregnant. Despite their young age most were aware of the different tests available. The results further indicate most of the students had a positive attitude towards prenatal testing in general.

Two thirds of the students would consider undergoing NIPT. This positive attitude is in line with prior research. For example, Lewis et al. (2014) found that their respondents were overwhelmingly positive regarding the introduction of NIPT. They concluded that uptake is likely to be high, and even includes women who currently decline screening as well as those who will use the test for information only. Lewis et al. also recommended pre-test counseling as essential to ensure that women undergoing the test understand the implications of the test result. van Schendel and colleagues (2015) conducted focus groups as well as individual interviews and found that most of the participating women and men had a very positive attitude towards NIPT when the test was introduced. At present, decision-making about prenatal testing is partly influenced by fear of miscarriage (invasive test) – a disadvantage NIPT does not have – and by uncertainty of test results (FCT), which is highly reduced with NIPT. However, van Schendel et al. (2015) raised some concerns about high levels of participation in NIPT and they stressed the importance of facilitating an informed choice.

More than two thirds of the students in the present study stated that it would be a major issue to have a baby with Down syndrome. However, it is known that the overall level of knowledge about the consequences of Down syndrome is low or at least varying. A Swedish study of 105 pregnant women and 104 partners showed that both women and partners had varying or low levels of knowledge about medical, cognitive and social consequences of Down syndrome (Ternby et al. 2015). Only 23 % of their sample reported having received information about what it means to have a child with Down syndrome. The finding that 23 % of the students in the present study indicated personal experiences of someone with chromosome aberration or a severe disease as influential in their hypothetical decision to undergo prenatal testing was unexpectedly high. Such a motivation suggests at least some reflection on their decision at this relatively young age. However, it is not known if the students’ motivation to undergo prenatal testing is to prepare themselves for having a disabled child, as previously has been shown (Hurford et al. 2013), or to prepare for the possibility of terminating the pregnancy.

Regarding the question about how to receive information concerning prenatal testing, surprisingly few students indicated the Internet as a desired source. Most reported preferring verbal information, and many indicated preference for a separate visit to the doctor or midwife at the antenatal clinic. These results highlight the importance of personal contact to inform about prenatal testing. A German study showed that people in upper social classes, women, persons in a stable relationship, and natives search the Internet for health related purposes to a higher extent compared to lower social classes, men, singles and persons with a migration background (Nölke et al. 2015).

Most participants in the present study wanted to know if the baby has Down syndrome, and an even larger majority wanted to know about more severe chromosome aberrations. In contrast, it seemed less important to find out the gender of the fetus. These results are lower compared to a study from the USA, where 58 % of the mothers and fathers wanted to learn about the gender of the baby before birth (Shipp et al. 2004). In Sweden, the clinical experience from prenatal testing is that many parents-to-be want to know the gender of their baby. The findings in the present study are that about 37 % of the students stated that they wanted to know the gender of the fetus, which is lower than in a recent Swedish study of 1003 pregnant women in which 50 % stated that they wanted to know the gender of their baby (Sahlin et al. 2016). Perhaps, the question and importance of gender have had to make way for more important issues in the context of prenatal testing.

The risk of a twenty-year-old woman having a baby with Down syndrome is 1:1526. Most of the students in the present study seemed to have a realistic picture of the actual risk of having a baby with Down syndrome. However, many students overestimated the risk in relation to the actual age-related factor. This might be because risk perception to a large extent is affected by one’s feelings (Loewenstein et al. 2001) and by individual background (Slovic et al. 2004). Furthermore, there are considerable effects on risk perception as a result of the language used when communicating. Melas et al. (2012) performed a study with a sample similar to the one in this study. They found that teenagers perceived technical jargon and words such as “rare” and “abnormal” as worrying. Understanding how each individual interprets information concerning a genetic condition or a risk estimate is challenging. There is a need for a conscious effort to use empirically-supported strategies for risk information in clinical practice. Differences in perception related to numeric risk formats were present among the teenagers in the Melas et al. (2012) study.

Most of the students in the present study reported that it would be the partner who influences their decision about prenatal testing. Almost half indicated that family and friends would influence the decision. This is in line with previous research showing that the partner and other close persons play an important role in decision-making (Garcia et al. 2008). Family, such as one’s parents, may be even more significant in decision-making when asking these young people.

In the study by Melas et al. (2012), no gender differences were identified in their sample’s responses to a questionnaire was answered. In the current study, only two questions yielded gender dependent differences. A greater percentage of women indicated a more favorable attitude about ultrasound examination and a greater percentage reported they would make the decision about prenatal testing themselves. Thus, there were few gender differences, and as noted in the next section, multiple univariate tests may have yielded significant differences that are due to chance. The relative lack of gender differences is consistent with the results of a recent qualitative study by Van Schendel et al. (2014). Those researchers did not find any striking differences between women and men in attitudes towards NIPT.

### Study Strengths and Limitations

Strengths of the study include that the questionnaire was short and distributed to all students in the selected grades. The questionnaire was only distributed in one high school, however, which reduces the generalizability of the results. The selected high school was located in the center of Stockholm, the capital of Sweden. The students were mostly Swedes with a high socio-economic status. In Sweden, the national health care system is mostly tax financed and the antenatal care is easy to access and free of charge. These factors may further limit generalizability.

None of the items on the questionnaire asked participants about previous or current pregnancies, which would certainly have had a great impact on their responses. The students were between 16 and 18 years old, with a mean age of 17 years. In Sweden, about 1 % of the babies are born to women 19 years old or younger, and 2.4 % of female adolescents become mothers annually (The National Board of Health and Welfare 2015). Guided by those figures it is assumable that very few participants were pregnant at the time of data collection.

A number of univariate tests were conducted to assess gender differences and differences for students who would or would not undergo NIPT. They were done without a correction (e.g., Bonferroni) to control for familywise error rate. Although this is considered acceptable in an exploratory study, there is a greater likelihood that some of the significant findings are due to chance.

Finally, the results are based on responses to a hypothetical situation. They may or may not reflect participants’ actual attitudes and behaviors.

### Practice Implications

Information about prenatal testing in general and risk information in particular is a great challenge in antenatal care. A large amount of information is expected to be delivered during early pregnancy. To some extent, young people, high school students, have knowledge about prenatal testing and are able to express their preferences for information. Lectures and information about genetic conditions and the possibilities of detection of those conditions may be delivered in high school in order to allow sufficient time for reflection prior to the first pregnancy. This study highlights the importance of personal meetings with health care providers who are knowledgeable about prenatal screening and testing in order to provide individuals with relevant information. The information is complex and there may be a need to discuss not only methods and conditions which are tested for, but also ethical issues. In addition to the healthcare providers mentioned in the questionnaire used in this study, genetic counselors are ideally positioned to communicate this type of information given their professional preparation and roles in healthcare.

### Research Recommendations

An intervention comprised of a curriculum of lectures, discussions and seminars designed for high school and university students that includes plain facts about genetic conditions, chromosomal aberrations, prenatal testing, and associated ethical issues should be developed and tested. Short and long term effects on attitudes, knowledge and decision making about prenatal testing should be evaluated. Additional studies with older samples, individuals who have and have not had a pregnancy, and those with and without personal experiences with genetic conditions are also warranted.

## Conclusion

The majority of the high school students was positive towards prenatal testing and most were positive towards NIPT. Further, information was desired by the vast majority before decision making about NIPT, and most of the students preferred verbal information and to a lesser extent information via the Internet. There were few gender differences in attitudes and preferences.
